# The interaction between acute emotional states and executive functions in youth elite soccer players

**DOI:** 10.3389/fpsyg.2024.1348079

**Published:** 2024-03-25

**Authors:** Simon Knöbel, Henrietta Weinberg, Florian Heilmann, Franziska Lautenbach

**Affiliations:** ^1^Faculty of Sport Science, Chair of Sport Psychology, Leipzig University, Leipzig, Germany; ^2^Sport Psychology, Institute of Sport Science, Humboldt-Universtität zu Berlin, Berlin, Germany; ^3^Movement and Sport Psychology, Institute for Sport Science, Friedrich-Schiler-University Jena, Jena, Germany; ^4^Movement Science Lab, Institute of Sport Science, Martin-Luther University Halle-Wittenberg, Halle, Germany

**Keywords:** executive functions, elite youth soccer, diagnostics, emotion, affect, talent development

## Abstract

**Introduction:**

Executive functions (EFs) are relevant for game performance in soccer and have been investigated in previous research. However, emotions are a well-known performance factor in sport competitions, which may affect performance by means of EFs. The diagnostic of EFs has mainly been performed disregarding the potential impact of emotional states. Thus, we aimed to initially analyze interaction between emotional states and EFs in two studies with male youth elite soccer players.

**Methods:**

In the first study, 105 players (*M*age = 14.97) completed computerized tasks assessing inhibition, cognitive flexibility and working memory. In the second study, 92 players (*M*age = 15.17) performed adapted and validated tests of inhibition (*n* = 45) or cognitive flexibility (*n* = 47) in a soccer-specific setting (SoccerBot360). Emotional and affective states were assessed using the German Sport Emotion Questionnaire and self-assessment manikins.

**Results:**

For the computerized tasks, results showed a significant negative correlation between switch costs accuracy and tension, *r* = 0.28, indicating lower error rates with higher levels of tension. In contrast, in the SoccerBot360 we found significant positive correlations for response time and tension (no-switch: *r* = 0.38; switch: *r* = 0.39) representing prolonged response times related to tension. Further, for soccer-specific inhibition, positive emotions were significantly positively correlated with response time (congruent: *r* = 0.32; incongruent: *r* = 0.32). Subsequent regression analyses also demonstrate that valence and arousal effectively explain variance in cognitive performance parameters under neutral conditions.

**Discussion:**

Accordingly, the ambiguity of the results suggests high variability in EF performance, affective and emotional states as well as a potentially moderating influence of other variables such as context and task difficulty. Thus, future cognitive diagnostic research should integrate assessments of emotional and affective states as these may contribute to situational fluctuations in EF performance.

## Introduction

Since soccer has been characterized as a strategic sport in which players have to adapt to dynamically changing situations, executive functions (EFs) play a significant role in game performance (see reviews: Voss et al., 2010; Scharfen and Memmert, 2019a). In addition, high technical and tactical performance requires both, perceiving as well as processing relevant internal and situational external information (Smith et al., 2016). EFs are the basis of these cognitive processes that enable the integration of information into goal-directed action (Diamond, 2013). According to the Unity/Diversity model [presented initially by Miyake et al. (2000)], EFs can be divided into three core abilities: Inhibition (inhibitory control), cognitive flexibility (shifting), and working memory (updating), which are not entirely independent but can be distinguished from each other. In addition to physical and technical requirements, the characteristics and development of performance in soccer are determined by numerous (interacting) personal and external factors (Murr et al., 2018). Emotions have been shown to affect physical, perceptual-cognitive, and behavioral domains and are therefore relevant to performance (e.g., Lazarus, 2000; Jones, 2012), for example by influencing EFs (Lautenbach and Laborde, 2016). Existing theories provide different explanations and directions of interaction (Sweller et al., 1998; Mitchell and Phillips, 2007). In this context, disregarding emotional states can also be considered as a potential limitation in previous diagnostics of EFs (Lautenbach et al., 2016; Beavan et al., 2020). Thus, this study aimed to provide a first step by considering acute emotions within diagnostics of EFs and perform an initial analysis of associations among variables in two different samples of elite youth soccer players.

### Cognition in soccer

Research on cognitive skills is primarily based on two approaches (see Meta-Analyses, Voss et al., 2010; Kalén et al., 2021), namely the cognitive component skills approach (Nougier et al., 1991) and the expert performance approach (Ericsson, 2003). Whereas the cognitive component skills approach measures general cognitive functions predominantly assessed with standardized computer-based cognitive tasks and button press responses, the expert performance approach focuses on sport-specific perceptual-cognitive processes in corresponding settings (e.g., evaluate pictures or video material depicting a particular sport). There has been empirical evidence for both approaches, generally supporting the relevance of cognitive functions in soccer (e.g., Huijgen et al., 2015; Musculus et al., 2019). Therefore, both approaches were applied in the two studies conducted. The relevance of using sport-specific response modalities and stimuli in the scope of cognitive diagnostics has been emphasized as they are more likely to reflect expertise differences between different performance levels (see meta-analysis of Kalén et al., 2021). The application of both approaches within the research question allows a comparison of which emotional states occur in different settings of cognitive diagnostics and how they correlate with EFs in the respective context.

Besides an associated genetic determination of EF, existing sport-specific evidence indicates considerable interpersonal variability in cognitive diagnostics among athletes of different experience and performance levels (Verburgh et al., 2014; Huijgen et al., 2015) but also within cohorts of similar levels (Beavan et al., 2020; Vestberg et al., 2020). However, many practice-relevant issues and methodological approaches are challenging to implement because EFs combine various processes with different prerequisites and, thus, remain open or unexploited. Exemplarily, the diagnostics of EFs under consideration of potential confounders has been disregarded. Since soccer matches involve high pressure of a competitive situation, accompanied by the experience of positive and negative emotions (Vast et al., 2010), an isolated examination of EFs considerably limits the transfer of the results to the actual performance on the field (Van Maarseveen et al., 2018). However, situational factors such as motivational and emotional states are rarely established or considered in the diagnosis of EFS (Lautenbach et al., 2016).

### Emotions and executive functions

Neuroimaging studies have shown a functional overlap between frontal brain structures (e.g., prefrontal cortex) that support EF processes as well as processing of emotional stimuli (Gyurak et al., 2012, p. 2). While EFs are primarily referred to as top-down processes (Miyake and Friedman, 2012), emotions are experienced in a specific person-environment relationship that is determined by button-up and top-down processes. Relevant environmental stimuli are processed via attention and perception, while internal evaluation and judgment processes shape emotional responses (Smith and Lane, 2015). Theories focusing on the interplay of emotion and cognition assume that characteristically emotions are associated with changes in informational and cognitive processes such as attention, memory, planning, and problem solving (Smith and Lane, 2015). A general assumption is that emotional states can influence the receptivity to certain stimuli and information in such a way that selective perceptions of information arise (Rowe et al., 2007).

#### Defining and measuring emotions

At this point, it is necessary to refer to the distinction between emotions and interrelated constructs such as affect and moods. Affect is in accordance to Russell (2003) always present even though not continuously processed, and can be described on two psychophysiological dimensions that is valence (pleasant vs. unpleasant) and arousal (high vs. low). Thus, every emotional state can be represented as a combination of a certain valence and a certain level of arousal (e.g., circumplex model, Russel, 1980). Emotions are short-term affective states that can be traced back to a specific event. Besides, emotions can be verbalized, which allows categorization in discrete emotions such as anger, joy or anxiety. In contrast, moods do not always have a triggering event and are longer-lasting, less intense than emotions, and less susceptible to regulatory processes (Gross, 2008; Vaughan and McConville, 2021, p. 675). Overall, these constructs are often used interchangeably (Beedie et al., 2005; Tamminen and Bennett, 2017). This also applies to commonly used measurement instruments (e.g., POMS; McNair et al., 1971), that are not strictly used to assess their underlying constructs or cover parts of those constructs instead of the entire domain (Ekkekakis, 2012).

Generally, a distinction between positive and negative emotions is established in the literature following the two dimensions of valence (e.g., Solomon and Stone, 2002; Smith and Lane, 2015). However, this classification seems to be questionable for discrete emotions, as not all emotions clearly fit in one of these categories [see borderline cases in Lazarus (2000)]. In this context also cultural and linguistic aspects have to be considered regarding the definition of distinct states. A commonly used measure for emotions in the sport context is the Sports Emotions Questionnaire by Jones et al. (2005). The current study used the validated German version of the Sports Emotions Questionnaire (SEQ-d; Wetzel et al., 2020) which deviates partially from the English version. While the original questionnaire measures emotions on five dimensions (i.e., anxiety, dejection, excitement, anger and happiness), the dimensions were reduced in the German version due to cultural differences (i.e., positive emotions, negative emotions and tension). Thus, tension represents and independent dimension that seems to lie between positive and negative emotions. In the German questionnaire validation, tension positively correlated with negative emotions, suggesting low correspondence with the nearby English translation “excitement,” which has an unequivocal positive understanding (Wetzel et al., 2020). At this point, it must be considered that not only linguistic interpretations of the items can differ, but also the individual evaluation of the situation. Within the model of facilitative and anxiety (Jones, 1995) it is suggested that the appraisal of symptoms triggered by emotions such as anxiety is central to the influence on performance. Accordingly, it is possible that athletes feel nervous and experience corresponding psychophysiological symptoms associated with high arousal, but perceive these as facilitative in performance situations. Thus, increased tension or negative and positive emotions and its interpretation can have different effects on performance, which is also central to Hanin’s (2000) model of individual zones of optimal functioning.

In addition to measuring discrete emotions, affect is often assessed. For example, using visual analog scales or via self-assessment manikins (e.g., SAM, Bradley and Lang, 1994). Since the SAM is a language-free measure presenting pictures of facial expressions, language or translation issues can be avoided. Therefore, the instrument was used in the present study alongside the SEQ-d to obtain a better understanding of the interplay between affect and emotion, as well as to precisely differentiate their impact on cognitive outcome variables. The consideration of appropriate measures for affect, mood and emotions has been highlighted by Ekkekakis (2012, p. 322) stating that it should depend on “whether the goal is to assess a specific, narrowly defined state (or a set of distinct states) or broad dimensions that are theorized to underlie a global domain of content (such as mood or core affect).” We therefore refer to emotions, but also consider affect as a component of emotions and moods in line with suggested theoretical models [see overview in Lautenbach (2024)].

### Cognitive load theory

The Cognitive Load Theory (Sweller et al., 1998) is based on the assumption of an individual’s limited working memory capacity, which can be impaired internally by the characteristics of the input to be processed or externally, by the way the material is presented. Cognitive load refers to the resources tied up in working memory by the execution of a cognitive task and are, therefore, not available for other processes. Accordingly, affect represents an extraneous cognitive load, which ties up a certain amount of the resources required for information processing in working memory. These resources can no longer be used for the actual task (Sweller et al., 1998; Mitchell and Phillips, 2007). This is explained by affect-related but task-irrelevant thoughts, which burden the cognitive system and, thus, relevant processes such as attention (Mitchell and Phillips, 2007; Vaughan and McConville, 2021). It is assumed that both positive and negative affect are indirectly proportional to EFs performance. In other words, the higher the arousal of the affect, the lower the cognitive performance, due to a greater need for executive control. However, this prediction is subject to further internal and external factors, such as task difficulty or expertise (Sweller et al., 1998; Mitchell and Phillips, 2007). Thus, the prediction particularly refers to tasks that require high demands on individual’s executive resources. If the task itself is not challenging, the resources tied up by affective states may not be needed at all and do not necessarily lead to reduced performance. With respect to expertise it is assumed that working memory capacity, central to the hypothesis, can be expanded through experience (Plass and Kalyuga, 2019). Consequently, trained individuals could handle and process higher cognitive loads, so that extraneous loads would be less likely to impair cognitive task solving.

#### Mood-as-Information Theory

The Mood-as-Information Theory does not consider affect as an extraneous load but as a relevant source of information for perceiving and processing stimuli (Schwarz and Clore, 1983). The theory differentiates between positive and negative moods. Positive moods are associated with the absence of danger or threat, which is assumed to lead to reduced attention as well as automated and more heuristic processing (Mitchell and Phillips, 2007; Gabel and McAuley, 2018). In contrast, negative mood is related to threats or problems in the environment resulting in more careful and focused cognitive processing (Mitchell and Phillips, 2007). Accordingly, because the optimal performance of EFs involves both complex cognitive processing and associated attentional processes, positive mood impairs EFs, whereas negative mood is predicted to increase EFs.

### Empirical evidence

Previous research outside the sports context has demonstrated mixed results regarding the influence of emotion, mood and affect on cognitive performance^1^ depending on the measured functions and the task used as well as the particular emotion. With regard to the different concepts of emotion, mood and affect, it should again be noted that the terms are used interchangeably or interrelated in most studies. Investigating the effects of positive moods on EFs, Phillips et al. (2002) showed impairment in shifting which provides evidence for the cognitive load theory and Mood-as-Information Theory. At the same time, however, improved creative performance on a fluency task was observed. From these results, the authors concluded that the influence of positive moods on cognitive performance is not generalizable and may also be related to other factors, such as the intrinsic motivation elicited by the task at hand. Similarly, for negative mood states findings report impairment in working memory performance (Allen et al., 2014) which is in line with cognitive load theory. However, also improvements were shown for response inhibition rather reflecting the predictions made by Mood-as-Information Theory (e.g., Pessoa et al., 2012). Referring to these findings and based on their results that anxiety impairs EFs in contrast to anger, Shields et al. (2016) argue that it is necessary to differentiate between negatively valenced states as not all have the same effects on EFs. The authors then conclude that not only the valence of the affective state (i.e., negative vs. positive) plays a role but also the related level of arousal and motivation (Shields et al., 2016).

Consistent with the mixed results in previous research, the effects of experienced emotions (e.g., frustration) have been demonstrated to promote intraindividual differences in EF performance among school children (Pnevmatikos and Trikkaliotis, 2013). Similarly, Gabel and McAuley (2018) reported increased variability of cognitive performance in affective situations (e.g., performance contexts) in an adult sample from the general population.

Few studies have addressed interactions between distinguishable emotional states or affects and EFs in sports. Research in sports primarily addresses emotions and their effects on cognitive processes such as attention or concentration and competitive athletic performance (e.g., Vast et al., 2010; McCarthy et al., 2013). In this context, existing evidence primarily relates to anxiety or stress (Lazarus, 2000; Woodman et al., 2009). The interaction is described based on subjective optimal and suboptimal zones of the affective state for performance (e.g., Individual Zone of Optimal Functioning; Hanin, 2000). Accordingly, an emotion’s functionality depends on the individual appraisal of the situation and the accompanying subjective evaluation, influenced by available resources for coping (Lazarus, 2006). Concerning the core EFs, Vaughan and McConville (2021) reported a positive relationship between positive emotions and performance of EF in terms of higher accuracy and lower latencies. At the same time, they found a significant correlation between negative emotions, higher latency, and fewer errors. Both correlations were moderated by athletic expertise in terms of higher positive affect and better performance in EF tasks shown for higher-level athletes. These results show that positive and negative emotions can promote accuracy performance, however, have different impact on response times.

### The present study

Evidence suggests EFs correlates with acute emotional states and that these states can influence the performance of EF. The direction of influence is still debated (e.g., Vaughan and McConville, 2021). Whereas the Cognitive Load Theory (Sweller et al., 1998) assumes that cognitive performance declines regardless of the affect that occurs, the Mood-as-Information Theory (Schwarz and Clore, 1983) predicts improved performance concerning negative emotions and a decrease in EF performance in positive states. Accordingly, this study examines correlations of emotions, affect and core EFs in elite youth soccer players. In doing so, we follow the approach of similar studies on the initial analysis of the connection between two constructs that are potentially interrelated (e.g., EFs and physical-motor abilities, Scharfen and Memmert, 2019b, 2021). With regard to the common approaches of cognitive diagnostics, both the classical cognitive tasks of the cognitive component approach as well as adapted versions of these tests for a soccer-specific^2^ setting, including a soccer-specific motor response (i.e., passing a ball; Musculus et al., 2022) within the expert performance approach, were applied independently for data collection. Therefore, the methods conducted will be regarded concerning correlations between the performance in the respective tasks and the collected emotional and affective states.

We hypothesize correlations between the dimensions of emotions classified based on the German sports-emotion questionnaire (i.e., negative, positive, tension) and core EFs. Given equivocal assumptions in existing theoretical models and previous findings as well as the commonly mentioned contextual features limiting generalizability (e.g., Shields et al., 2016; Vaughan and McConville, 2021), we do not specify the hypotheses on the direction of effects. Also, following the existing theoretical assumptions, we do not differentiate effects with respect to the different EFs (inhibition, cognitive flexibility, working memory).

Further, in a more exploratory fashion, we aimed to examine the variance of response times in the respective EF tasks as an indicator of stable cognitive performance (McKinney et al., 2020; Martin-Niedecken et al., 2023) in relation to the reported emotions. Based on previous results, we expect a positive correlation between emotional states and the individual variance of cognitive performance (e.g., Gabel and McAuley, 2018), reflected by response times. Accordingly, we assume that positive and negative emotions contribute to fluctuations and intraindividual differences due to physiological (e.g., dopamine release; Van der Stigchel et al., 2011) or psychological adaptations (e.g., self-regulation; Pnevmatikos and Trikkaliotis, 2013) evoked by emotions. In this context it has been suggested that EFs alter in situations with different environmental and contextual factors (Pnevmatikos and Trikkaliotis, 2013, p. 258).

## Methods

### Participants

In Study 1, we recruited 105 male soccer players from a local youth soccer academy with a mean age of 14.97 years (*SD* = 1.84), of which were all male and belonged to the U13, U14, U15, U16, U17, and U19 youth teams.

In Study 2, a total of 91 male soccer players were recruited from the youth academy of a German first-division soccer club. All players belonged to the U15, U16, U17, and U19 youth teams (*M*_age_ = 15.1 years, *SD* = 1.4). The unequal number of players in both studies results from the different sizes of the respective teams of the clubs. Please see the detailed characteristics of participants for both studies in Supplementary Table B1. At the time of data collection, all participating teams played at the top level of their respective age group. To the best knowledge of the participating clubs, participants did not have any diagnosed behavioral, learning, or medical conditions that might influence cognitive abilities. Only healthy and fit players took part in the tests on a voluntary basis. Prior to the investigations, written informed consent was obtained from the participants and their legal guardians. Both studies were carried out in accordance with the Declaration of Helsinki and approved by the ethics committee of Leipzig University (ethic number: 2020.11.17_eb_69).

### Material

In Study 1, the three main components of EFs according to Miyake and Friedman (2012), were measured with computerized tasks via Inquisit Lab 5 (Millisecond Software LLC, 2018). The test series included the 3-back task, flanker task, and number-letter task in a counterbalanced order.

In Study 2, the flanker task and the number-letter task were applied in the SoccerBot360 (SB) as the validation of the n-back task measuring working memory has not been completed at this time (Knöbel and Lautenbach, 2023). The SB is a circular training device with a diameter of 10 m that provides for a 90-m^2^ field in a 360° environment surrounded by a 32- segment wall, each segment 1-m wide and 2.5-m high, serving as a projection area for the training content and against which played balls can be kicked. Six high-definition projectors generate the training content. An integrated high-speed camera enables recording parameters such as response time, processing time, and accuracy. The playing’ field’s ground consists of artificial grass (see Supplementary Figure A1). The SB therefore offers a setting for soccer players that is more representative of the specific context. This refers to the fact that players can perform tasks in combination with motor movements that are also relevant in the game (i.e., ball control, passing). In addition, the on-screen environment can be designed in a game-like manner (e.g., stadium, spectators).

### Computerized and soccer-specific inhibition

**Figure 1:**
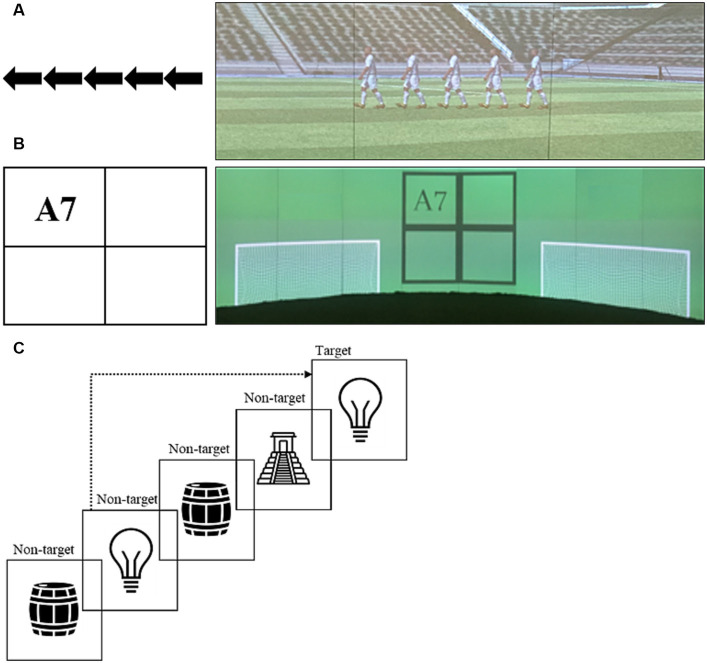
Presentation of stimuli in the general, computerized tasks applied in study one [**(A)** flanker task, **(B)** number-letter task, **(C)** n-back task] and the soccer-specific versions for the flanker task (inhibition) and number-letter task (cognitive flexibility).

Inhibition was assessed with an arrow version of the flanker task (FT; Eriksen and Eriksen, 1974). In the computerized task, five arrows were presented on a 17-inch screen. The middle arrow is the target stimuli, and the test person has to decide whether it points to the left or the right. To distract the test person from the direction of the target, other arrows, the flankers, are displayed at the same time. For congruent trials, all arrows point in the same direction (Figure 1A), whereas for incongruent trials, the target and the flankers are in opposite directions. If the target pointed to the left, the participants had to respond with the “E” button. If the target pointed to the right, the “I” button had to be pressed. The task contained four practice and 72 test trials, divided into two blocks. One-third of the test runs were incongruent (see Krenn et al., 2018). To measure the function of inhibition with the flanker task, response times for correct responses in milliseconds (ms) and accuracy of responses in percent (%) were collected for congruent and incongruent trials. Additionally, to describe the impact of the incongruence of stimuli, the flanker effect was calculated from the differences in mean response times between congruent and incongruent trials. Reliability of the task was assessed via Cronbach’s α for the respective conditions (congruent: 0.93, incongruent: 90).

For the soccer-specific flanker task, five soccer players were presented from the side (Figure 1A). Similar to the computerized flanker task, the target player was in the middle, with two distracting players on each side. Depending on the direction in which the target player faced, participants were asked to kick the ball into the left or right goal that served as equivalent to the response button in the computerized task presented on either side of the players (see Supplementary Figure A1). The soccer-specific flanker task consisted of 54 trials (36 congruent, 18 incongruent, see Musculus et al., 2022). The reliability of the task was assessed via Cronbach’s α. High values for the respective conditions within the task could be determined (congruent trials: 0.97; incongruent trials: 0.96).

### Computerized and soccer-specific cognitive flexibility

Cognitive flexibility was assessed using a version of the number-letter task as adapted from Rogers and Monsell (1995) by Miyake et al. (2000). It contains a 2×2 matrix that displays sequentially clockwise a pair of characters in one of the four boxes (see Figure 1B for soccer-specific task). Thereby, the combination of either even (2, 4, 6, 8) or odd (3, 5, 7, 9) numbers and either vowel (A, E, I, O) or consonants (G, K, M, R) has to be assessed regarding the position in the matrix. In the upper boxes, only the letter is of interest and the digit functions as a distractor that is irrelevant for the answer, vice versa in the lower boxes. In the computerized version, the participant had to respond with a button press of “E” for consonants and even numbers and of “I” for vowels and odd digits. For the soccer specific version, the participant had to pass the ball into the left goal for consonants and even numbers, and in the right goal for vowels and odd number.

To familiarize the players with the task, two non-switch blocks were introducedinitially, in which they started with the number modality for the first block followed by the letter modality for the second. Non-switch means that stimuli appear either in the upper or the lower row and therefore only the letters or the numbers have to be responded to. After that, a combined block incorporating both conditions was presented, prompting participants to attend to switches between upper and lower rows and adjust their responses accordingly. This combined block then includes the non-switch and switch conditions and represents the final task.

For the computerized version, 24 practice trials were conducted for each condition, followed by 24 combined trials and then 64 test trials, including 32 switch and 32 no-switch trials. Response time in milliseconds (ms) and accuracy scores of responses in percent (%) for the switch and no-switch trials were assessed. The difference between the no-switch and switch trials for response time and accuracy reflects the switch costs. The lower the switch costs, the higher the performance in cognitive flexibility. Calculation of Cronbach’s α indicates a high reliability of the test (no-switch: *α* = 0.85, switch: *α* = 0.87).

For the soccer-specific version, participants first completed a total of 24 practice trials divided into three blocks: eight non-switch runs in each of the letter and number rows, followed by eight combined trials. The percentage of correct answers had to be at least 75% in during practice trials, otherwise, the block had to be repeated to ensure participants understood the task. Concerning reliability, high values were determined for switch trials (*α* = 0.92) and no-switch trials (*α* = 0.94) for the soccer-specific task.

### Computerized working memory

The 3-back task was used to examine the working memory performance (Jaeggi et al., 2010). Eight stylized black/white pictures of everyday objects are displayed sequentially for 500 ms each with a pause of 2,500 ms in between. The participants had to decide if the picture they currently see is the same as the one presented three trials before (see Figure 1). If so, then they had to press the button “A.” If not, then they did not have to respond at all. We conducted a series of 46 stimuli, including 18 target trials after a previous practice run with 23 stimuli. Response time in milliseconds (ms) and response accuracy in percent (%) have been collected. The lower the response times and the higher the accuracy scores, the higher the working memory performance.

### Emotional and affective states

In both studies, acute emotional states were collected with the SEQ-d adapted by Wetzel et al. (2020) from the original English questionnaire by Jones et al. (2005). The questionnaire contains three dimensions: “positive emotions,” “negative emotions” and “tension.” The 13 items are measured on a 5-point Likert scale from 0 to 4, which rates the grade of approval to emotion-related adjectives. Cronbach’s Alpha shows the reliability of the dimensions calculated based on the collected data from Study 2. “Positive emotions” contain the items “joyful,” “pleased,” “cheerful” and “happy” (*α* = 0.76), while negative emotions” include “angry,” “furious,” “disappointed,” “subdued” and “dejected” (*α* = 0.62). The dimension of “tension” includes the items “nervous,” “excited,” “tense” and “uneasy” (*α* = 0.84).

Valence and arousal of participants were recorded before the EF tasks using the Self-Assessment Manikin (SAM) to complement the emotional states. The SAM is a non-verbal assessment using pictorial representations to “measure the pleasure, arousal, and dominance associated with a person’s affective reaction to a wide variety of stimuli” (Bradley and Lang, 1994, p. 49). Objectivity and reliability of the SAM could be proven and it is widely used as it is non-verbal and therefore can be applied irrespective of the subjects’ age or background (Morris, 1995). For valence, we applied one row of five pictograms, including a 9-point scale from 1 (“unpleasant”) to 9 (“pleasant”). For arousal, the lowest point of the scale was “calm” (1) up to “excited” (9).

### Procedure

Data for Study 1 were collected in the fall of 2020. EF data from this sample have been previously reported in Heilmann et al. (2022). However, no reports or analyses on affect and emotions have been published in the previous article as these data were not integral to the primary research question on the age-dependent development of EFs. Despite overlapping reported EF data, this article addresses distinct questions and presents unpublished additional findings (in accordance with COPE guidelines; Urbanowicz and Reinke, 2018). The experiment was conducted at the youth training center facilities of the participating club and lasted approximately 40 min for each player. First, participants were welcomed, signed an informed consent form, and then filled out the SEQ-d and the SAM. Afterwards, they performed the cognitive tasks in a randomized order.

Data for Study 2 were collected between June and November of 2021. The experiment was conducted in the youth soccer academy of the participating club and lasted approximately 60 min for each player. Players were randomly assigned to one of the cognitive tasks – either the number-letter task or the flanker task. Players completed a short standardized warm-up program to reduce the risk of injuries before performing the respective task. Following the warm-up and immediately before the soccer-specific cognitive task in the SB, participants were asked to fill out SEQ-d and the SAM. In both studies, participants reported their emotional states immediately before the start of the cognitive test, without the induction of specific emotions.

### Data preparation

For the first study, overall, five participants had to be excluded from all analyses because they did not have complete data sets for any of the cognitive tasks or incomplete questionnaires. Thus, complete datasets were available for 100 participants. Subsequently, the data within each task were analyzed separately and different exclusion criteria were applied as filter (please find detailed description for both studies in Supplementary material, section C).

In the second study, measuring inhibition and cognitive flexibility with adapted tasks in the SB, it should be reiterated that, unlike in Study 1, each player performed only one randomly assigned cognitive task. In addition, two players had to be excluded because of missing questionnaire data (one in the flanker task and one in the number-letter task). Accordingly, the 90 players with complete data sets available are divided into two tasks whose data were processed and analyzed independently (see Supplementary material, section C).

### Data analyses

For both studies, all data were analyzed using SPSS statistics, version 27. The level of significance was set at *p* = 0.05 for all analyses. Shapiro–Wilk test was used for testing for normal distributions. Not all cognitive variables were normally distributed, as assessed by Shapiro–Wilk’s test (*p* < 0.005). For a detailed description of the results of normal distribution and the procedure for identifying and dealing with outliers please see Supplementary material (Section C).

To examine contextual overlap or differences between measures, correlations between the emotion dimensions of the SEQ-d (i.e., positive emotions, negative emotions, tension) and affect assessed by the SAM dimensions (i.e., valence and arousal) were calculated. In Study 1, correlations of the questionnaire data were performed across all participants with at least one complete cognitive test, as the experimental design included all three tasks without subdivisions into different groups. Conversely, Study 2, conducted correlational analyses within each task, as the experimental design included only one task per participant (i.e., flanker task or number-letter task). Accordingly, both tasks were performed by different players. Subsequently, for testing hypothesis 1, we calculated Pearson correlations between emotional states assessed with the SEQ-d as well as affect assessed with the SAM and the performance parameters of the respective tasks. For the flanker task measuring inhibition, analyzed parameters were mean response times and accuracy for congruent and incongruent trials, along with the difference between their means of representing the flanker effect. Similarly, for the number-letter-task measuring cognitive flexibility, mean response times and accuracy for switch trials and non-switch trials as well as differences between the means (Switch Costs) were retrieved. For measures of working memory with the n-back task in Study 1 we looked at response times of correct target trials and overall accuracy.

Furthermore, to examine hypothesis 2 whether variance in cognitive performance on the computerized and soccer-specific tasks is related to emotional states, we determined the intraindividual standard deviation, that is, the standard deviation of the single trials averaged per subject. For working memory, the deviations between the single response times of every correctly answered target trial were determined. For inhibition and cognitive flexibility, the variance of response times for every trial in the respective conditions was added and correlated with the reported emotions and affect.

## Results

Statistical analyses indicated the same pattern of results when outliers were included, and thus, all analyses are reported, including the outliers. Since not all data were normally distributed, we performed non-parametric correlational analyses using Spearman’s Rho. Descriptive data for the questionnaire data of both studies and correlations between the applied measures are presented in Table 1. The descriptive statistics for all response times and accuracy values for the computerized and soccer-specific tasks are shown in Table 2.

**Table 1:** Descriptive data on the German sports emotion questionnaire (SEQ-d) and control variables arousal and valence assessed with the self-assessment manikin (SAM).

	Descriptive statistics				Correlations r_s_ between measures				
	*M*	*SD*	Min	Max	SEQ-d PE	SEQ-d NE	SEQ-d Tension	SAM Valence	SAM Arousal
Study 1: *N* = 100									
SEQ-d PE	2.68	0.64	1	4					
SEQ-d NE	0.49	0.69	0	3.2	−0.251*				
SEQ-d Tension	1.36	0.93	0	3.5	0.009	0.206*			
SAM Valence	7.42	1.31	2	9	0.316**	−0.199*	−0.154		
SAM Arousal	3.02	1.78	1	8	−0.102	0.195	0.440**	−0.206*	
Study 2: number-letter task (*n* = 35)									
SEQ-d PE	2.32	0.81	0	4					
SEQ-d NE	0.31	0.32	0	1	−0.400*				
SEQ-d Tension	1.06	0.82	0	3	0.036	−0.238			
SAM Valence	7.46	1.22	4	9	0.499**	−0.393*	−0.183		
SAM Arousal	2.86	1.45	1	7	−0.192	0.085	0.499**	−0.509**	
Study 2: flanker task (*n* = 44)									
SEQ-d PE	2.38	0.77	0.75	4					
SEQ-d NE	0.27	0.43	0	1.8	−0.124				
SEQ-d Tension	0.98	0.82	0	3.25	0.147	0.172			
SAM Valence	7.59	1.18	5	9	0.207	−0.184	−0.291		
SAM Arousal	2.91	1.6	1	8	−0.154	0.486**	0.565**	−0.409**	

^**^The correlation is significant at 0.01 level (twofold). ^**^The correlation is significant at 0.05 level (twofold). SEQ-d, Sport-emotions questionnaire (German version); PE, positive emotions; NE, negative emotions; SAM, Self-assessment Manikins.

**Table 2:** Descriptive statistics on the computerized task for inhibition, cognitive flexibility, and working memory and soccer-specific tasks for inhibition and cognitive flexibility.

Study: Task	Descriptive statistics			
	*M*	*SD*	Min	Max
Study 1: Number–letter task (*n* = 90)				
RT, no-switch trials (ms)	1009.63	213.89	590.47	1629.42
RT, switch trials (ms)	1442.35	319.39	838.05	2416.70
RT, switch costs (ms)	432.72	233.73	−143.34	1234.78
Accuracy, no-switch (%)	94.63	5.52	71.88	100.00
Accuracy, switch (%)	87.17	8.53	65.63	100.00
Accuracy, switch-costs (%)	7.47	7.56	−6.25	31.25
Variance, no-switch trials (SD)	407.84	132.03	146.15	646.57
Variance, switch trials (SD)	454.88	122.34	141.45	781.24
Study 2: Number–letter task (*n* = 35)				
RT, no-switch trials (ms)	1408.54	334.29	874.38	2110.16
RT, switch trials (ms)	1529.98	369.86	978.68	2298.75
RT, switch costs (ms)	121.44	146.12	−71.12	509.04
Accuracy, no-switch (%)	94.28	6.94	75.00	100.00
Accuracy, switch (%)	91.63	6.24	75.00	100.00
Accuracy, switch costs (%)	2.65	6.38	−14.29	17.86
Variance, no-switch trials (SD)	294.72	132.27	112.96	609.63
Variance, switch trials (SD)	311.93	124.48	97.74	537.87
Study 1: Flanker task (*n* = 98)				
RT, congruent trials (ms)	444.00	80.04	310.83	726.04
RT, incongruent trials (ms)	469.19	81.86	333.40	780.92
RT, flanker effect (ms)	25.18	24.73	−51.84	101.10
Accuracy, congruent trials (%)	98.28	2.37	87.50	100.00
Accuracy, incongruent trials (%)	96.34	4.87	83.33	100.00
Accuracy, flanker effect (%)	1.93	5.06	−8.33	16.67
Variance, congruent trials (SD)	74.38	35.64	27.95	179.56
Variance, incongruent trials (SD)	92.64	51.37	35.34	242.99
Study 2: Flanker task (*n* = 44)				
RT, congruent trials (ms)	946.27	154.52	712.97	1250.50
RT, incongruent trials (ms)	961.60	161.84	707.88	1295.11
RT, flanker effect (ms)	15.33	28.40	−34.33	96.43
Accuracy, congruent trials (%)	99.68	1.07	94.44	100.00
Accuracy, incongruent trials (%)	99.62	1.85	88.89	100.00
Accuracy, flanker effect (%)	0.06	2.20	−5.56	11.11
Variance, congruent trials (SD)	100.32	39.26	52.62	210.39
Variance, incongruent trials (SD)	102.87	34.34	49.27	198.81
Study 1: *n-back* task (*n* = 98)				
RT, target trials (ms)	837.53	317.14	349.17	1615.00
Acc, overall (%)	35.72	17.26	−3.33	79.00
Missed targets (%)	30.50	12.02	0.0	61.11
False alarms (%)	18.80	14.60	0.0	96.43
Variance, target trials (SD)	300.17	202.66	1.5	808.85

### Study 1: correlations between emotion and affect

We found significant negative correlations between valence and negative emotions (*r* = −0.199, *p* = 0.047) as well as negative and positive emotions (*r* = −0.251, *p* = 0.012). Further, valence was positively correlated with positive emotions (*r* = 0.316, *p* = 0.001) and negatively correlated with arousal (*r* = −0.206, *p* = 0.040). In addition, we found significant positive correlations between the emotion dimension tension and negative emotions (*r* = 0.206 *p* = 0.040) and tension and arousal (*r* = 0.440, *p* < 0.001). All correlational results for emotion and affect across all participants of Study 1 are reported in Table 3.

**Table 3:** Correlations between emotional states, affect and cognitive performance in the computerized task for, inhibition, cognitive flexibility and working memory (Study 1) and in the soccer-specific tasks for inhibition and cognitive flexibility (Study 2).

Variable	Emotion dimensions (SEQ-d)	Flanker task			Number-letter task			n-back task
		Congruent	Incongruent	Flanker effect	No-switch	Switch	Switch costs	Target trials
Study 1								
Response time	Positive emotions	0.057	−0.077	−0.151	0.036	0.054	0.025	0.022
	Negative emotions	−0.025	0.045	0.134	0.073	−0.048	−0.158	−0.064
	Tension	0.107	0.069	−0.008	−0.034	−0.042	0.022	−0.030
	Valence	0.146	0.100	−0.158	0.147	0.159	0.047	0.092
	Arousal	−0.069	−0.070	0.065	−0.007	−0.040	−0.050	−0.055
Accuracy	Positive emotions	0.153	−0.052	0.101	−0.085	0.099	−0.127	−0.019
	Negative emotions	−0.076	−0.054	0.006	−0.121	−0.094	−0.007	0.044
	Tension	0.019	−0.023	0.061	−0.044	0.165	−0.287**	0.012
	Valence	0.141	0.090	−0.054	−0.004	−0.015	0.090	−0.005
	Arousal	−0.039	−0.145	0.131	0.053	0.029	−0.020	−0.073
Study 2								
Response time	Positive emotions	0.327*	0.328*	0.019	−0.206	−0.208	−0.067	
	Negative emotions	0.014	−0.040	−0.080	−0.047	−0.073	−0.135	
	Tension	0.066	0.030	−0.094	0.387*	0.391*	0.025	
	Valence	0.439**	0.476**	0.218	−0.138	−0.179	−0.069	
	Arousal	−0.276	−0.337*	−0.281	0.511**	0.485**	−0.035	
Accuracy	Positive emotions	0.129	−0.105	0.161	−0.067	0.012	0.062	
	Negative emotions	0.023	0.188	−0.085	0.252	0.073	−0.079	
	Tension	−0.216	−0.042	−0.148	−0.043	−0.265	−0.223	
	Valence	0.145	−0.062	0.150	−0.360*	0.029	0.393*	
	Arousal	−0.294	0.327*	−0.415**	0.122	−0.133	−0.293	

^**^The correlation is significant at 0.01 level (twofold). ^**^The correlation is significant at 0.05 level (twofold).

### Study 1: correlation between cognitive performance, emotions, and affect

For inhibition performance parameters, no significant correlations could be detected. For cognitive flexibility, correlational analyses revealed a significant negative correlation between tension and accuracy switch costs (*r* = −0.287, *p* = 0.006). For working memory performance parameters, no significant correlations were shown for the dimensions of emotions or valence and arousal. Besides the reported correlations, no other significant results were found. All correlational results regarding cognitive performance in study 1 are presented in Table 3.

### Study 1: variance in cognitive performance

For Study 1, no significant correlations were found with respect to the standard deviation in response times between the trials of the cognitive computer tasks.

### Study 2: correlations between emotion and affect

For the soccer-specific tasks in Study 2, all correlational analyses were performed within the respective task, as the experimental design within the project included only one task (i.e., flanker task or number-letter task) for each participant. Accordingly, both tasks were performed by different players.

With regard to affect and emotion dimensions for the players performing the flanker task (*n* = 44), we found significant positive correlations between negative emotions and arousal (*r* = 0.486, *p* < 0.001). In addition, arousal correlated positively with tension (*r* = 0.565, *p* < 0.001) and negatively with valence (*r* = −0.409, *p* = 0.006).

Analyses of measures within the number-letter task (*n* = 35) showed significant negative correlations between positive and negative emotions (*r* = −0.400, *p* = 0.017) and between negative emotions and valence (*r* = −0.393, *p* = 0.019). Further, a significant negative correlation could be detected between valence and arousal (*r* = −0.509, *p* = 0.002). In contrast, significant positive correlations were found for valence and positive emotions (*r* = 0.499, *p* = 0.002) as well as arousal and tension (*r* = 0.499, *p* = 0.002). All correlational results for affect and emotion dimensions for the computerized tasks in Study 1 and the soccer-specific tasks in Study 2 are presented in Table 1.

### Study 2: correlation between cognitive performance, emotions, and affect

Correlational analyses for cognitive performance in the soccer-specific flanker task to measure inhibition revealed significant positive correlations between mean response times and positive emotions in the congruent (*r* = 0.327, *p* = 0.030) and the incongruent condition (*r* = 0.328, *p* = 0.030). Likewise, response times were positively correlated with valence (congruent: *r* = 0.439, *p* = 0.003; incongruent: *r* = 0.476, *p* = 0.001). Further, significant negative correlations were shown between arousal and response times in the incongruent condition (*r* = −0.337, *p* = 0.025). In addition, arousal was positively correlated with response accuracy in the incongruent condition (*r* = 0.327, *p* = 0.030) and negatively correlated with the flanker effect for accuracy (*r* = −0.415, *p* = 0.005).

For cognitive flexibility, significant positive correlations were detected between tension and response times in the no-switch (*r* = 0.387, *p* = 0.022) and switch condition (*r* = 0.391, *p* = 0.020). In addition, arousal showed significant positive correlations with response times (no-switch: *r* = 0.511, *p* = 0.002; switch: *r* = 0.485, *p* = 0.003). Concerning accuracy, we found a significant negative correlation with valence in no-switch trials as well as a positive correlation for valence and accuracy switch costs. All correlations involving EF performance are presented in Table 3.

### Study 2: variance in cognitive performance

The data from the soccer-specific flanker task showed a significant correlation between the standard deviations in response times in the congruent condition and valence (*r* = 0.359, *p* = 0.017). In contrast, for cognitive flexibility, significant positive correlations were detected between variance in response times for arousal and no-switch trials (*r* = 0.506, *p* = 0.002) as well as switch trials (*r* = 0.422, *p* = 0.012).

### Additional analyses

In order to follow the multifaceted results of the correlational analyses, we subsequently conducted regression analyses for the cognitive variables that were significantly correlated with affect or emotions. For this purpose, we calculated a multiple linear regression model in which the affective dimensions valence and arousal as well as the three emotion dimensions (positive, negative, tension) were tested as predictors of cognitive performance. The correlations between the measurements (SEQ-d and SAM) do not indicate multicollinearity and the non-normal distribution of some cognitive parameters was retained and not transformed for analysis (see Schmidt and Finan, 2018).

For Study 1, the results of the regression analysis indicated that the affective and emotional states (valence, arousal, positive emotions, negative emotions, tension) did not predict accuracy switch costs, *R*^2^ = 0.11, *F*(5,86) = 2.079, *p* = 0.076. For Study 2, the regression results for inhibitory performance indicated that the emotional and affective measures explained 33% of the variance in response times on congruent trials, *R*^2^ = 0.33, *F*(5,38) = 3.742, *p* = 0.007. The affective dimension of valence showed a significant effect (*β* = 0.394, *p* = 0.010). For incongruent trials, the model explained 34% of the variance in response times, *R*^2^ = 0.34, *F*(5,38) = 3.915, *p* = 0.006. Again, only valence showed a significant effect (*β* = 0.399, *p* = 0.009). The model was also significant for response accuracy on incongruent trials, explaining 26.3% of the variance, *R*^2^ = 0.26, *F*(5,38) = 2.712, *p* = 0.034. The emotion dimension of tension (*β* = −0.509, *p* = 0.003) as well as the affective dimension of arousal (*β* = 0.445, *p* = 0.010) showed significant effects. In contrast, the model for the flanker effect for accuracy was not significant, *R*^2^ = 0.24, *F*(5,38) = 2.420, *p* = 0.053.

For cognitive flexibility, regression results indicated that the model explained 41.9% of the variance in response times on noswitch trials, *R*^2^ = 0.41, *F*(5,29) = 4.191, *p* = 0.005. However, a significant effect was only found for arousal (*β* = 0.685, *p* = 0.003). Similar patterns were observed for the response times in switch trials with 40.4% of the variance explained by the predictors, *R*^2^ = 0.40, *F*(5,29) = 3.937, *p* = 0.008. Here, significant effects were found for arousal (*β* = 0.615, *p* = 0.007) and positive emotions (*β* = −0.333, *p* = 0.047). For accuracy, we detected no significant results.

Finally, the predictors (positive and negative emotions, tension, valence and arousal) did not predict intraindividual variability in response times in the inhibitory task. However, for cognitive flexibility, results indicate that 35.7% of the variance across noswitch trials were explained by emotion and affect, *R*^2^ = 0.35, *F*(5,29) = 3.216, *p* = 0.020. A significant effect was only detected for arousal (*β* = 0.722, *p* = 0.003). In contrast, the model was not significant for switch trials, *R*^2^ = 0.30, *F*(5,29) = 2.488, *p* = 0.054.

## Discussion

The present studies addressed whether acute emotional states are related to EF performance in male elite youth soccer players. For this purpose, EFs were measured in two samples of different clubs using two methodological approaches. While the first study investigated general cognitive abilities with computerized tasks (i.e., inhibition, cognitive flexibility, working memory), the second used adapted validated versions (i.e., inhibition, cognitive flexibility) in a soccer-specific setting. Comparing results of both studies, we found significant correlations between the reported emotional and affective states and performance parameters of the conducted cognitive tasks in accordance with our first hypothesis. However, correlational patterns differ substantially between the studies and tasks, so several rationales need to be considered when interpreting the results. Furthermore, we conducted additional regression analyses to explore whether our affective and emotional measures were significant predictors of cognitive outcomes.

### Tension, arousal and EF performance (Study 1 and 2)

For the first study, correlation analyses revealed a significant negative correlation between switch costs for number-letter task accuracy and the SEQ-d dimension of tension. This indicates that a higher perceived tension is related to lower mistakes in switch trials in comparison to non-switch trials, and, thus, improved shifting performance. These findings could not be replicated in the second study. However, the affective dimension of arousal was positively correlated to response accuracy in incongruent trials and negatively correlated to the flanker effect in response accuracy, pointing to reduced interference effects similar as Study 1. In addition, we found significant positive correlations between tension as well as arousal and response times within the switch and no-switch condition of the number-letter task in the second study. In other words, the higher the participants’ perceived tension, the more time they needed to response, thus, the lower their cognitive flexibility. In contrast, a negative correlation between arousal and response times in incongruent trials was shown in the soccer-specific flanker task (Study 2), indicating that tension might contribute to faster response times and better selectivity in the inhibitory task.

Focusing first on the ambiguous results with regard to the emotion dimension tension, a closer look at the dimension and the term seems necessary to understand the results. To our knowledge, this is the first empirical study that has applied the German version of the SEQ. Consequently, it might be a first approach to consider the connotation or meaning of the dimension of *tension*, which might be inconclusive. In this context, comparisons of the SEQ-d and the German POMS (profile of mood states; Biehl et al., 1986) to test convergent validity for the German-speaking region revealed weak correlations between *tension* of the SEQ-d and *dejection/anxiety* but also *zest for action* of the POMS (Wetzel et al., 2020). This illustrates the difficulty of classifying *tension* on an emotion dimension as it can be interpreted both positively and negatively which possibly also leads to an ambivalent understanding of the queried items.

The correlational analyses in the first study indicated a positive relationship between tension, negative emotions and arousal. This suggests that tension had a rather negative connotation, aligning with observations from the validation of the German questionnaire (Wetzel et al., 2020). Following this line of argument, our results from Study 1 could be interpreted in favor of the Mood-as-Information Theory predicting better cognitive performance and more effective processing with negative emotions accompanied by higher arousal, as we found that higher levels of tension were positively related to better shifting performance for accuracy. In contrast, findings from Study 2 rather support the Cognitive load theory stating that affective states generally demand cognitive resources and result in a decline in EF performance. Overall, our results strengthen the argument that differentiations must be made between negatively valenced states depending on their corresponding arousal (Shields et al., 2016).

Considering the analyses for the second study, it is noticeable that tension does not correlate with negative emotions, however correlates with arousal similarly to Study 1. Thus, the results suggest that there could be partial conceptual overlaps between the two measurements (i.e., arousal and tension) by assessing physical components, whereas the dimension tension of the SEQ-d might presents a more differentiated assessment of arousal than the SAM. Regarding the EF tasks, higher tension was associated with impaired performance (i.e., response times in the number-letter task) while the correlations concerning arousal indicated better performance (i.e., lower response times and higher accuracy in the flanker task). These contrasting results between the studies and between the players in the respective tasks in Study 2 may also have been caused by different levels of tension and arousal. Players in Study 1 reported higher levels of tension and arousal than in Study 2. Arousal in terms of physiological and psychological activation, which also seems to be reflected from the items of the tension scale of the SEQ-d, could be an indicator of general alertness and readiness to solve the task (e.g., McConnell and Shore, 2011). Thus, the higher intensity of tension and the association with negative emotions found among participants in the first study may have contributed to more attentive and effective processing of the stimuli. This could possibly explain the reduction of the error difference between the conditions represented by the accuracy switch costs (Study 1). In contrast, performance parameters of the soccer-specific number-letter task in Study 2 seemed to be negatively affected by tension which might be attributed to lower intensity. Additionally, also the appraisal of the situation could contribute to these differing patterns. While arousal was highly correlated with negative emotions in the flanker task, this could not be demonstrated for the players within the number-letter task. According to this, a generally more positive appraisal of the situation among the players of the number-letter-task could have resulted in lower arousal and less intense physiological symptoms that were not facilitative for task performance (Jones, 1995). However, the low internal consistency of the dimension negative emotions potentially questions the reliability of the SEQ-d questionnaire and must also be noted in these considerations. The low value could possibly also be attributed to testing under neutral conditions. While the questionnaire was answered prior to an actual competition as part of the validation process (Wetzel et al., 2020), the studies in which we applied the questionnaire did not include a competitive context. The absence of a competitive situation may also account for the relatively small effect sizes observed between measures in our correlational analyses. Thus, in relation to the individual appraisal it is possible that the player’s answers to the queried items were inconsistent or rather arbitrary because their agreement to the single items was rather based on their personal well-being before the start of testing than on specific events within the test situation. This interpretation is reinforced by the subsequent multiple regression analyses, which showed that the dimension of tension does not yield significant effects on response times related to cognitive flexibility. However, the notable effects of arousal suggest that affect, which is consistently present and not necessarily attributed to specific events (e.g., Russell, 2003), is a more suitable predictor of response times under neutral conditions. In contrast, both tension and arousal explain a substantial amount of variance for response accuracy during incongruent trials of the inhibitory task. This underscores the diverse potential impacts of emotions, which can be facilitative or debilitative for different parameters of cognitive performance (Vaughan and McConville, 2021).

The different levels of tension and their perception in both samples of youth soccer players possibly also led to different behavioral tendencies. This influence was elaborated by Smith and Lane (2015) in terms of fear and anger, which are both valenced negatively and entail high arousal but provoke contrary behaviors. Whereas anger is associated with eliciting approach behavior, fear tends to lead to avoidance behavior. There might have been higher response times in the second study for the number-letter task related to tension because the players wanted to avoid mistakes in the first place and therefore, needed more time to decide for the target goal (speed-accuracy trade off, e.g., Fitts and Peterson, 1964).

Moreover, the contrasting correlation results in study two could also be explained by the different requirements of the assigned tasks. While the rules for the number-letter task change constantly and thus, require permanent adjustment of focus, the response condition for the flanker task is consistent (i.e., pass the ball into the direction that the middle player faces). Therefore, on the one hand, it can be assumed that the number-letter-task presents a higher task difficulty which has been shown to be related to lower attentional control (see Grahek et al., 2020) and results in higher response times. On the other hand, high arousal and tension coupled with lower task difficulty may directed player’s focus on reacting as quickly as possible in the flanker task, which promoted their accuracy in the incongruent condition and thus minimized the flanker effect.

### Positive emotions, valence, and EF performance (Study 2)

In addition, we also found significant positive correlations for positive emotions as well as valence and response times for congruent and incongruent trials in the flanker task. Thus, response times seem to be prolonged when higher positive emotions are reported. Here, we found similar patterns across the tasks in Study 2, as the valence-related correlations in the number-letter task indicated lower accuracy in no-switch trials accompanied by increased switch costs. These results align with Mood-as-Information Theory predicting a decline in cognitive abilities for positive emotions. Similarly, they support the Cognitive load theory, according to which any affect contributes to a reduction in cognitive performance. Rowe et al. (2007) reported similar results for the flanker task, stating that that although a positive affect has a broadening effect on attention (e.g., broaden-and-build theory, Fredrickson, 2001) it can also restrict selectivity and thus inhibitory control. Follow-up regression analyses also indicate that affect better explains response times in the flanker task under neutral conditions, given that only valence showed significant effects as predictor. Nevertheless, it is worth noting that positive emotions exhibited significant effects on response time in no-switch trials, with a negative regression coefficient suggesting that positive emotions tended to forecast a reduction in response times. Contrary to the models previously discussed, this aligns with the facilitator theory (Isen, 1987, 1999), suggesting that individuals in positive mood states possess increased cognitive resources and broader attention, fostering enhanced creativity and cognitive flexibility. Furthermore, this could again point to variances in emotional intensity among the players engaged in the respective tasks. Contrary, effects of valence or positive emotions have not been found for the computer task data, indicating contribution of potential confounding factors such as the perception of the task environment (see Grahek et al., 2020). Even though, participants’ positive emotions are descriptively higher prior to the computer task in comparison to the soccer-specific task, we found in a previous study that the soccer specific task has been perceived to be more fun than the computer task (Musculus et al., 2022). Thus, it could be that during the task itself, positive emotions might have increased due to the soccer-specific task including the specific motor action and therefore, was performed less attentive as predicted by the Mood-as-Information Theory, resulting in higher reaction times and lower accuracy in no-switch trials. However, this is speculative in nature and such confounding factors (e.g., task difficulty, utility expectancy; Grahek et al., 2020) should be considered in future research.

### Affect and EF variance (Study 2)

In general, our results indicate that affective and emotional states appear to have greater effects on performance parameters in the soccer-specific tasks potentially due to the discussed environmental and contextual conditions. Additionally, this could be related to the required motor response due to the different measurement methods. The SB requires a more complex movement and thereby, more extensive physical preparation and synchronization in comparison to a button press in the computerized task. The motor response’s complexity directly influences simple reaction times (e.g., memory-drum theory, Henry and Rogers, 1960). In this context, it has been shown that emotional states that are accompanied by physiological arousal, affect “physical and cognitive subcomponents of performance” differently (Woodman et al., 2009; p. 170). These differential effects may also help explain the findings regarding variance in cognitive performance.

For the soccer-specific number-letter task, our analyses revealed positive correlations between variance in response times in switch and no-switch trials with arousal as well as predictive effects of arousal for no-switch trials. Accordingly, increased arousal seems to provoke higher variability in response times reflecting lower stability of performance. Thus, emotional and physiological arousal appear to have possibly different effects between the single trials of the task. In other words, the effects of arousal seem to be rather arbitrary, so that it can favor both faster as well as slower responses. Again, the results differ in the flanker task, in which it is the affective dimension of valence that correlates with the variance of response times in congruent trials. This could also reflect the different requirements and complexities of the tasks discussed before. Overall, these results are in line with our second hypothesis and support previous findings that variability of cognitive performance is increased by affective experiences (e.g., Brose et al., 2012; Gabel and McAuley, 2018). Further, they could also provide an explanation for why large differences in performance within samples with similar performance and experience levels have been observed in previous diagnostics of EFs. The occurrence of these variances has mainly been attributed to isolated diagnostics of single executive components and to interpersonally varying abilities within a complex performance structure (Beavan et al., 2020; Vestberg et al., 2020). At the same time, it has been suggested that emotional and motivational states may contribute to the variance (Beavan et al., 2020). Our results support this assumption and again highlight the consideration of these factors in cognitive diagnostics as well as the specific integration of different conditions to gain insights into which situations favor or impair cognitive performance (e.g., Pnevmatikos and Trikkaliotis, 2013).

### Limitations

The conducted studies present some limitations that should be considered when interpreting the results. First, it is eminent to state that no emotions were induced or elicited experimentally. The descriptive data in Table 2 shows that levels of tension, positive and negative emotions are comparably low to other studies that use anxiety or stress inductions or collect emotional states related to competition (e.g., Vast et al., 2010; Pessoa et al., 2012). Therefore, the intensity of emotions can be assumed to be fairly low as the subjects performed the tests under *neutral* conditions. In addition, the studies in both settings only applied emotionally neutral stimuli, as opposed to studies in which the stimuli were emotionally-laden (Lautenbach et al., 2016; Shields et al., 2016).

The choice of two different samples should also be mentioned in these considerations. A within-subject across all participants design might be a promising alternative to the two separately conducted studies. However, the duration of the experimental procedure as well as the cognitive load for each subject would then have been much higher. Inevitably, cognitive fatigue would have had to be controlled, and the duplication of tests in a short period of time would probably have had a direct impact on motivation and performance. The sample sizes were further based on the available and injury-free players of the youth teams of the respective academies. Due to these requirements, the overall sample size was limited. In addition, the players in the second study were assigned to only one task, which led to a considerably smaller number of players per test than in the first study. Relatedly, with regard to the generalizability of the data, also common characteristics of the samples need to be considered. All participants belonged to youth academies of professional soccer clubs and thus, are characterized as elite youth athletes. Correspondingly, a certain experience in dealing with performance situations can be assumed. Vaughan and McConville (2021) previously identified sports expertise as a moderator variable with regard to the influence of affect on EF. To verify this possible difference to the general population, a comparison with a non-(competitive) sports control group would have been necessary.

### Future directions

Notwithstanding the limitations mentioned above, the results point to the relevance of assessing and including emotional states in the diagnosis of EFs. The correlations found under neutral conditions show potential relations of emotional and affective states on different parameters of cognitive performance. This results in added theoretical and practical value for the assessment of EF performance in connection with the manipulation of emotional states. The deliberate manipulation of emotions into different states could provide further insights into the mechanism behind the influence of emotions and EFs (see also Mitchell and Phillips, 2007; Grahek et al., 2020) and vice versa [see, e.g., cognition-emotion interactions by Dreisbach (2023)]. Hence, to specify sports psychological training and interventions with respect to cognitive training and emotions, further research is needed. In this process, emotional reactivity as a trait-like characteristic of an individual could also play an important role. Exemplary, findings on increased variability of EF performance were attributed to emotional reactivity reflecting the “rapidity, intensity and duration of affective responses” (Gabel and McAuley, 2018; p. 863). Contrary to what is postulated by Mood-as-Information Theory or Cognitive load theory, a general impairment or improvement of executive performance in negative or positive affective states may not be applicable, which is also supported by our results. Thus, the additional recording of emotional reactivity and other personality traits (e.g., emotional intelligence; Vaughan et al., 2019) as well as the above-mentioned confounding factors (e.g., task difficulty, perception of task environment; see Grahek et al., 2020) could represent other important aspects of the influence of emotions on EF performance.

## Conclusion

In two separate studies, we examined whether affective or emotional states correlate with core EFs in youth elite soccer players and predict cognitive outcomes. Both underlying frameworks, the Cognitive load theory and the Mood-as-Information Theory cannot explain the findings satisfactorily. Whereas Study 1 indicated partly improved cognitive performance during higher tension, Study 2 could not reproduce these findings and indicated contrary patterns. A possible explanation for those ambivalent findings could be the dimension tension of the SEQ-d by itself, which is not specified in detail and thereby, not traceable to a concrete construct. Additionally, Study 2 found a relationship between positive emotions as well as valence and increased response times in the flanker task, which aligns with both theoretical approaches. The regression analyses offer deeper insights into the interplay of affect, emotions, and EFs. Our findings suggest that emotional and affective measures can significantly explain a substantial part of the variance in cognitive performance, with affect primarily serving as a predictor under neutral conditions.

The various associations of affective and emotional states on EFs illustrate potentially wide-ranging and nongeneralizable interactions, susceptible to situational and individual confounders. Although the correlation analyses do not depict any causal relationships, the results point to diverse practical and sports psychological implications. These include a consideration of the cognitive effort that must be performed and the task measuring it, as well as the valence of the emotional state and the accompanying arousal. Consequently, the results of both studies highlight the importance of affective and emotional states to be controlled for within cognitive diagnostics.

## Data availability statement

The raw data supporting the conclusions of this article will be made available by the authors, without undue reservation.

## Ethics statement

The studies involving humans were approved by Ethics Advisory Board, Leipzig University. The studies were conducted in accordance with the local legislation and institutional requirements. Written informed consent for participation in this study was provided by the participants’ legal guardians/next of kin.

## Author contributions

SK: Data curation, Formal analysis, Investigation, Writing – original draft, Writing – review & editing. HW: Data curation, Formal analysis, Investigation, Writing – review & editing. FH: Conceptualization, Project administration, Supervision, Writing – review & editing. FL: Conceptualization, Methodology, Project administration, Supervision, Writing – review & editing.

## Footnotes

^1^Cognitive performance is used interchangeable with EF performance throughout the manuscript and refers to the core EFs and not higher order processes.

^2^Soccer-specific means that the tasks were conducted in a setting where participants were standing and responding to stimuli with a soccer-specific motor response, namely passing to goals (Musculus et al., 2022).
