# Supplementary material for: The interaction between acute emotional states and executive functions in youth elite soccer players
Source: Front Psychol. 2024 Mar 25;15:1348079. doi: 10.3389/fpsyg.2024.1348079 (PMC10999690; doi:10.3389/fpsyg.2024.1348079)
Supplement: Supplementary file 1 [file Table_1.docx]

**Supplement Material**

**A) Presentation of Cognitive Tasks in SoccerBot360**

**Figure A.1.**

Presentation of flanker task (left) and number-letter-task (right) in the SoccerBot360 (study 2).


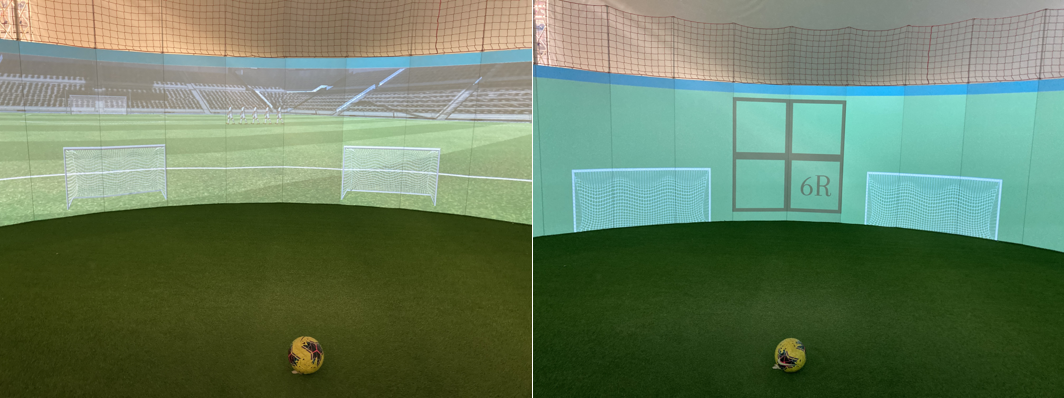


B) **Participants demographics**

|  | *M* | *SD* | *Min* | *Max* |  |
| --- | --- | --- | --- | --- | --- |
| **Study 1: *N* = 105** | | | | | |
| Age | 14.82 | 1.77 | 12 | 18 |  |
| Years of football | 9.93 | 2.40 | 2 | 14 |  |
| **Study 2: N = 92** | | | | | |
| Age | 15.18 | 1.46 | 13 | 18 |  |
| Years of football | 10.12 | 2.49 | 3 | 15 |  |

**Table B1.**

*Descriptive Data on Participants*.

***C)* Detailed Description on the Procedure for Data Preparation & Analyses**

***Data Preparation***

*Inhibition (Study 1 and 2)*

For the computerized flanker task for inhibition, a first filter^[[1]](#footnote-1)^ excluded all trials with incorrect responses (2.36%). In a second filter, all trials with response times lower than 200 ms or higher than 1.750 ms were excluded (0.03%) to account for extreme results (e.g., Lautenbach, Musculus et al., 2022). A third filter excluded response times ± 3 *SD* from the individual mean (1.95%). For the flanker task, two participants were excluded because of incomplete data sets. Thus, analyses for inhibition assessed with the computerized task include 98 participants.

The same procedure was applied for data preparation of the soccer-specific flanker task. However, the filters were slightly adjusted due to the different motor responses and leading to higher response times. The first filter excluded all trials with incorrect responses (0.33 %). For the second filter, response times lower than 400 ms or higher than 3.000 ms were identified as extreme results (Musculus, Lautenbach et al., 2022) and therefore, excluded (0.88%). A third filter excluded response times ± 3 SD from the individual mean (0.92%). One participant had to be excluded because of incomplete questionnaire data. Consequently, data for the soccer-specific flanker task included 44 participants.

*Cognitive flexibility (Study 1 and 2)*

For the computerized number-letter task used to measure cognitive flexibility, a first filter excluded all trials with incorrect responses (7.29%). The second filter (5.04%) and third filter (0.42%) were applied to account for extreme results. For the number-letter task, eight players were excluded because of incomplete data sets. An additional two players were excluded because they had an average accuracy (i.e., percentage of correct trials) of 70% or less (see Adrover-Roig et al., 2012; Lautenbach, Musculus et al., 2022). Thus, analyses for cognitive flexibility include 90 participants.

Similarly, for the soccer-specific number-letter task, the first filter excluded all incorrect responses (7.04%). The second filter was used with the same boundary values as in the flanker task (3.26%). Again, a third filter excluded response times ± 3 SD from the individual mean (1.17%). For the number-letter task, eleven players were excluded because they had an average accuracy of 70% or less and one participant because of incomplete questionnaire data. In total, analyses of the soccer-specific number-letter task include data from 35 participants.

*Working memory (Study 1)*

For the 3-back task measuring working memory, a filter was used to identify all missed target trials (30.42%). In addition, all responses to non-target trials (i.e., false alarms) were determined (18.87%), and the corresponding response times were excluded. Thus, the mean response time is calculated only on target trials that the participants answered correctly. Furthermore, a second filter was applied to exclude all response times that deviated ± 3 *SD* from the individual mean (0.0%). To calculate the overall accuracy in the first step, the percentage of correct answers to target trials and the rate of false alarms was determined. Then the percentage of correct target trials minus the percentage of incorrect answers for non-targets (i.e., false alarms) was calculated. Additionally, we investigated the percentage of missed target trials (see Knöbel & Lautenbach, 2023). Overall, two players had to be excluded because of incomplete data sets. Thus, analyses for working memory include a total of 98 participants.

**Data Analysis**

In Study 1, initially, all dependent variables were checked for normality and outliers. For computerized inhibition, only response times of the flanker effect were normally distributed. One outlier was detected for response times in the incongruent condition, one for accuracy in the congruent condition and two for incongruent accuracy values. Moreover, one outlier was detected for the flanker effect for the accuracy difference in both conditions represented by the flanker effect. For cognitive flexibility, response time in the no-switch condition were not normally distributed, whereas response times for switch condition and switch conditions had a normal distribution. Accuracy values of the respective conditions were not normally distributed. Five outliers (*SD* ± 2.5) were detected with regard to accuracy in the no-switch condition. For the *3*-back task measuring working memory, accuracy values were normally distributed, while response times did not show normal distribution. No outliers were detected for both parameters.

In Study 2, also all dependent variables were first checked for normality and outliers. For the flanker task in the SoccerBot360, response times for the incongruent and congruent conditions as well as flanker effect were normally distributed while accuracy parameters (i.e., percentage of correct trials) were not. Three outliers were detected with respect to accuracy in the congruent condition as well as two in the incongruent condition of the flanker task. In addition, five outliers were determined for the flanker effect of accuracy. Also, for the soccer-specific number-letter task, response times of no-switch trials and switch trials were normally distributed as well as switch costs which were not found for accuracy values. No outliers were detected in the number-letter task.

1. At this point, in order to understand the described procedure of data preparation and data analysis, a differentiation between filters and outliers must be noted. Different filters were applied to first identify extreme trials during data preparation and to exclude them before the analysis. The cleaned data were then checked again for statistical outliers in the subsequent data analysis. [↑](#footnote-ref-1)
